# *Mortierella alpina* CS10E4, an oleaginous fungal endophyte of *Crocus sativus* L. enhances apocarotenoid biosynthesis and stress tolerance in the host plant

**DOI:** 10.1038/s41598-017-08974-z

**Published:** 2017-08-17

**Authors:** Zahoor Ahmed Wani, Amit Kumar, Phalisteen Sultan, Kushal Bindu, Syed Riyaz-Ul-Hassan, Nasheeman Ashraf

**Affiliations:** 1 Plant Biotechnology Division, CSIR-Indian Institute of Integrative Medicine, Sanat Nagar, Srinagar, 190005 India; 2 Academy of Scientific and Innovative Research, CSIR-Indian Institute of Integrative Medicine, Canal Road, Jammu Tawi, 180001 India; 3 Instrumentation Division, CSIR-Indian Institute of Integrative Medicine, Canal Road, Jammu Tawi, 180001 India; 4 Microbial Biotechnology Division, CSIR-Indian Institute of Integrative Medicine, Canal Road, Jammu Tawi, 180001 India

## Abstract

*Crocus sativus* is the only plant species which produces apocarotenoids like crocin, picrocrocin and safranal in significant amounts. These compounds impart organoleptic properties to saffron (dried stigmas of *Crocus* flower) making it world’s costliest spice. *Crocus* apocarotenoids have tremendous medicinal properties as well. Effect of endophytes on *Crocus* apocarotenoid production and the molecular mechanism involved has not been reported so far. Here we studied the effect of an oleaginous fungal endophyte, *Mortierella alpina* CS10E4 on *Crocus* growth, apocarotenoid metabolism and tolerance to corm rot disease. The results demonstrated that there was a significant improvement in many morphological and physiological traits in endophyte treated *Crocus* plants including total biomass and size of corms, stigma biomass, number of apical sprouting buds, and number of adventitious roots. The endophyte also shifted metabolic flux towards enhanced production of apocarotenoids by modulating the expression of key pathway genes. Further, *M. alpina* CS10E4 enhanced tolerance to corm rot disease by releasing arachidonic acid which acts as conserved defense signal and induces jasmonic acid production in endophyte treated *Crocus* corms. This is first report on effect of a fungal endophyte on *Crocus* apocarotenoid metabolism and stress tolerance.

## Introduction

Although plants are sessile organisms, they are involved in intensive mutualistic associations with other organisms like microbes which help them interact with the surrounding environment^1^. These mutualistic microbes called endophytes are diversely localized and exert influence on many plant parameters like growth, metabolism and tolerance to biotic and abiotic stresses^1, 2^. Many endophytes especially the fungal endophytes are capable of producing bioactive natural products^3^. The microbes while harboured in different plant communities produce exclusive compounds of therapeutic value or they might as well produce the compounds which mimic some secondary metabolites produced by the host plant^4^. There are also reports where endophytes are shown to induce or at least enhance production of secondary metabolites by the host plant^5, 6^. Thus plant-endophyte interface provides an ecological marketplace for harnessing the potential of endophytes to produce compounds of therapeutic potential or exert their positive influence on plants to enhance the production of specialized metabolites of plant origin.

*Crocus* is an important plant used as a spice and medicinal crop since thousands of years^7^. *Crocus* finds a special place across plant taxa because of its ability to produce apocarotenoids like crocin, picrocrocin and safranal^8^. These compounds are used as coloring and flavouring agents in spice industry. Besides, these compounds are also known for their therapeutic potential^9^. This plant has remained outside the realm of genetic improvement because of its sterile nature. Poor agronomic practices and disease management together with lack of breeding approaches has led to declining trend in saffron production and quality. Moreover, biotechnological approaches have not helped much because transformation protocol has not been established. There are only a few reports where genes involved in the apocarotenoid biosynthetic pathway have been cloned and characterized^10–13^. Also a few transcription factors regulating the biosynthesis of these compounds have been identified^8^. However, none of these genes have been taken forward for transforming *Crocus* for enhanced production of apocarotenoids. This advocates the need to explore other possibilities for enhancing the production of *Crocus* apocarotenoids.

Since endophytes are known to have positive effect on plant secondary metabolites, it would be a wise choice to work on *Crocus* in this context and explore the role of endophytes in *Crocus* apocarotenoid metabolism. Work has been reported where bacterial endophytes have been isolated from *Crocus* and growth promoting activities of these endophytes have been documented^14^. Also fungal endophytes associated with *Crocus* corms have been isolated and their plant growth promoting properties reported^15^. In the present study we report effect of a fungal endophyte *M. alpina* CS10E4 on *Crocus* growth and secondary metabolite content. The endophyte positively affected many growth parameters like total biomass and size of corms, number of apical sprouting buds, and number of adventitious roots. Further, the endophyte enhanced overall accumulation of *Crocus* apocarotenoids by positively regulating expression of key pathway genes. It also enhanced biotic stress tolerance of *Crocus* to corm rot fungus by releasing arachidonic acid (AA) which is considered as an evolutionary conserved defense signal molecule that modulates plant stress signalling networks^16^. Also AA was shown to mediate its effect on corm rot through jasmonic acid pathway. This is to the best of our knowledge first report on effect of any fungal endophyte on *Crocus* apocarotenoid metabolism and its tolerance to corm rot.

## Results

### Isolation, characterization and growth promoting properties of *M. alpina* CS10E4

**Figure 1:**
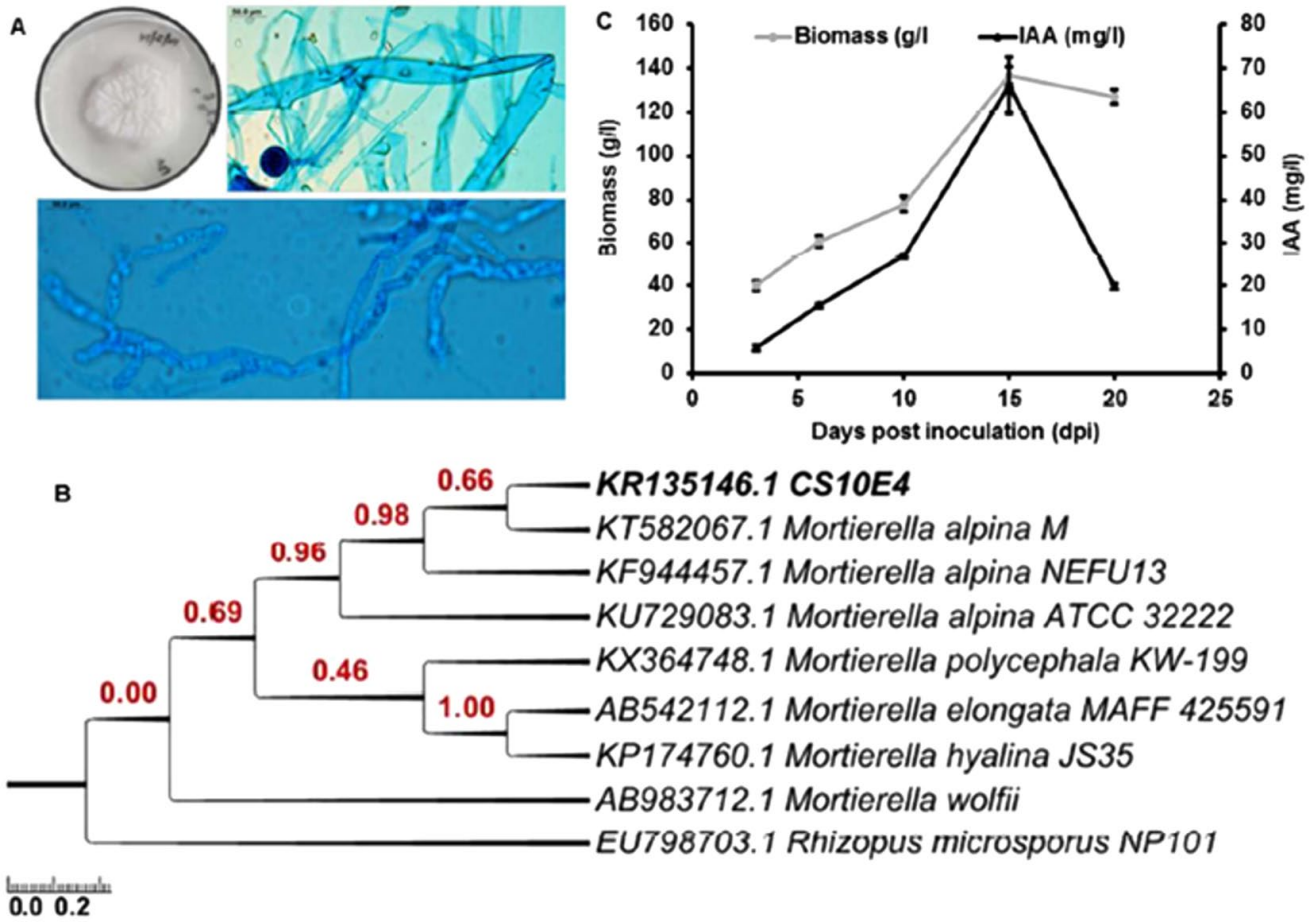
Morphology, molecular phylogeny and plant growth promoting property of the endophyte CS10E4 isolated from *Crocus sativus*. (**A**) Whitish colonies with zonate growth pattern on PDA plate. The mycelia are ceonocytic from which arise erect sporangiophores bearing terminal globose saporangia (**B**) the evolutionary history based on ITS1-5.8S-ITS2 ribosomal gene sequence was inferred by using the Maximum Likelihood method based on the Tamura-Nei model^30^. The tree is drawn to scale, with branch lengths measured in the number of substitutions per site. The analysis involved 9 nucleotide sequences and (**C**) time course accumulation of biomass and phytohormone (indole acetic acid) produced by the endophyte over a period of three weeks.

Previously 36 fungal endophytes were isolated from *Crocus sativus* corms in our lab among which *M. alpina* CS10E4 was the only zygomycete isolated at both stages of *Crocus* life cycle (*i.e*., dormant and vegetative stages)^15^. This prompted us to study the effect of *M. alpina* CS10E4 on growth and secondary metabolism of *Crocus*. Before evaluating its potential to enhance apocarotenoid content, *M. alpina* CS10E4 was tested for its growth pattern and plant growth promoting traits. The fungal colonies were fast growing producing a concentrate/zonate pattern on PDA plates. The mycelia were ceonocytic from which arise erect sporangiophores bearing terminal globose sporangia (Fig. 1A). Morphological and microscopic characteristics were in confirmation to that reported earlier for the species^17^. The ITS1–5.8S-ITS2 ribosomal gene sequence of CS10E4 showed highest similarity of 100% with *Mortierella alpina*. The phylogenetic positioning of CS10E4 is given in Fig. 1B.

Further, we observed that *M. alpina* CS10E4 produced significant amount of indole acetic acid (IAA) which increased till 15 dpi followed by a steep decline (Fig. 1C). At this time point we could record 66.2 mg/L of IAA from this fungus. The fungus was also found to be positive for siderophore production as seen in the form of orange halos on CAS agar plates (Fig. S1), therefore, confirming its growth promoting properties.

### Colonisation of *Crocus* corms with *M. alpina* CS10E4 and its effect on plant growth parameters

In order to assess the effect of *M. alpina* CS10E4 on *Crocus* growth and metabolism, the endophyte free corms were inoculated with the fungus and subsequently grown in green house under controlled conditions. The growth of the fungal endophyte and its successful colonization within the *Crocus* corms was confirmed by re-isolating the fungus from the corms at different growth stages viz 30 and 60 days after inoculation, flower primordia formation and flower emergence. The *Crocus* plants grown from the *M. alpina* CS10E4 inoculated corms were then studied for various growth parameters with more emphasis on corm characteristics, rooting system, flowering traits and chlorophyll content. We observed significant increase in total biomass and size of *M. alpina* CS10E4 treated corms as compared to the control ones. There was also an increase in the number of apical sprouting buds in endophyte treated corms. Further, we observed that the endophyte had an influence on rooting system of the plant. The number of adventitious roots had increased in case of the endophyte treated plants as compared to the control ones (Table 1, Fig. S2). Since the stigma part of the flower is the actual source of apocarotenoids, we calculated length and dry weight of stigmas collected from control and endophyte treated plants. It was observed that while there was no change in the length of stigmas, dry weight was higher in case of endophyte treated plants. We also observed more than two-fold increase in chlorophyll content of the endophyte treated plants as compared to the control ones (Table 1). Taken together these results indicate that *M. alpina* CS10E4 has a positive influence on overall growth parameters of *Crocus*.

**Table 1:** Effect of endophyte (CS10E4) on physiological and growth parameters of *Crocus sativus* plants.

Growth parameters	Endo−	Endo+	Significance (p < 0.05)
Size of corms	9.6 ± 0.83	10.5 ± 0.87	*
No. of sec. cormlets	3.3 ± 1.11	4.3 ± 1.62	ns
No. of apical buds sprouting per corm	2 ± 1.24	3.4 ± 1.56	*
No. of adv roots per corm	54.3 ± 8.71	64.87 ± 7.07	*
Biomass (g plant^−1^)	10.44 ± 2.33	12.29 ± 2.6	*
Length of stigmas (cm)	1.72 ± 0.3302	1.75 ± 0.2818	ns
Dry weight of stigmas (mg)	3.41 ± 1.219	4.395 ± 1.247	*
Total chlorophyll content (mg gFW^−1^)	0.97 ± 0.15	3.06 ± 0.31	*

(Endo−) are endophyte-free control and (Endo+) are the endophyte treated *Crocus* plants. Values are the means of 35 biological replicates ± S.D. Comparisons among means were carried out using Student T test at a significance level of p ≤ 0.05 using GraphPad InStat tm software (V2.05). Further, *indicates significance at p ≤ 0.05 and ns stands for non-significant.

### Effect of *M. alpina* CS10E4 on total secondary metabolite content

During the *Crocus* flowering season, flowers were collected from endophyte treated and control plants. The flower tissue was used to calculate the total phenolic, flavonoid and carotenoid content in these plants. We observed that *M. alpina* CS10E4 resulted in significant increase in total phenolics, flavonoid and carotenoid content in the host plant. The phenolic and flavonoid content increased from 37.7 ± 0.41 to 48.47 ± 0.55 and 47.13 ± 0.62 to 54.43 ± 0.65 respectively in endophyte treated plants as compared to control ones. Further, the total carotenoid content was almost doubled in endophyte treated plants (Table 2). The results, therefore, suggest that the endophyte positively regulates *Crocus* secondary metabolism.

**Table 2:** Estimation of total phenolics, flavonoid and carotenoid content in endophyte treated and control plants of *Crocus sativus*.

Secondary metabolites	Endo−	Endo+	Significance
Total Phenolic content (GAE/g of DW)	37.7 ± 0.41	48.47 ± 0.55	*
Total Flavonoid content (QE/g of DW)	47.13 ± 0.62	54.43 ± 0.65	*
Total Carotenoid content (µg/g of DW)	1.95 ± 0.25	2.95 ± 0.58	*

**(**Endo−) are the endophyte-free control and (Endo+) are the endophyte treated *Crocus* plants. Values are the means of 3 biological replicates  ±  S.D. Comparisons among means were carried out using Student T test at a significance level of p ≤ 0.05 using GraphPad InStat tm software (V2.05). *Indicates significance at p ≤ 0.05.

### Effect of *M. alpina* CS10E4 on apocarotenoid biosynthesis

**Figure 2:**
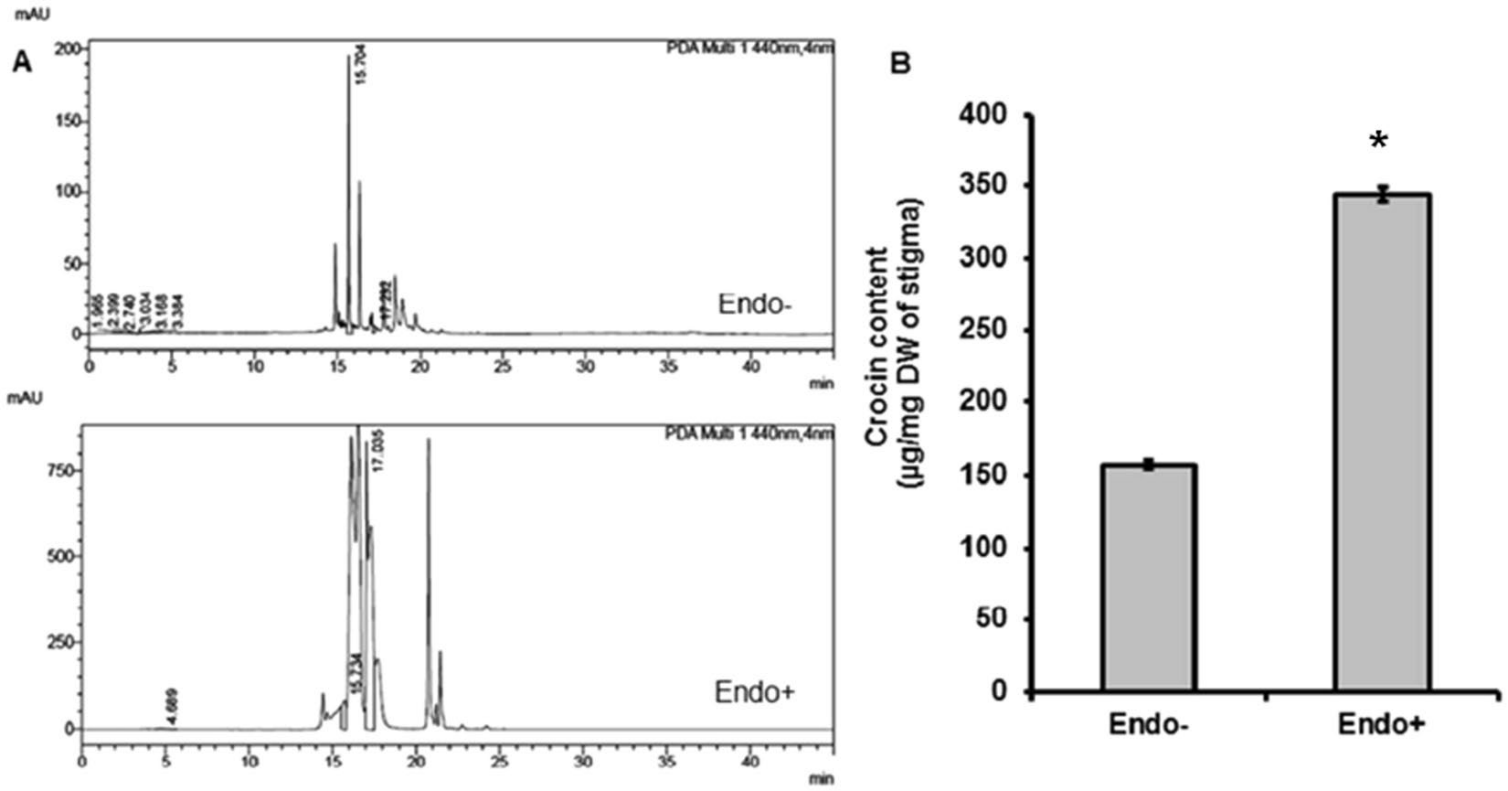
Effect of the CS10E4 on crocin content of saffron. (**A**) HPLC chromatograms showing crocin peaks in endophyte free (Endo−) and endophyte treated (Endo+) *Crocus* stigma samples (**B**) Bar diagram showing crocin content (µg/mg dry weight of stigma) in endophyte treated and control plants. Values are the means ± S.D. Comparisons among means were carried out using Student T test at a significance level of P ≤ 0.05 using GraphPad InStat tm software (V2.05).

**Figure 3:**
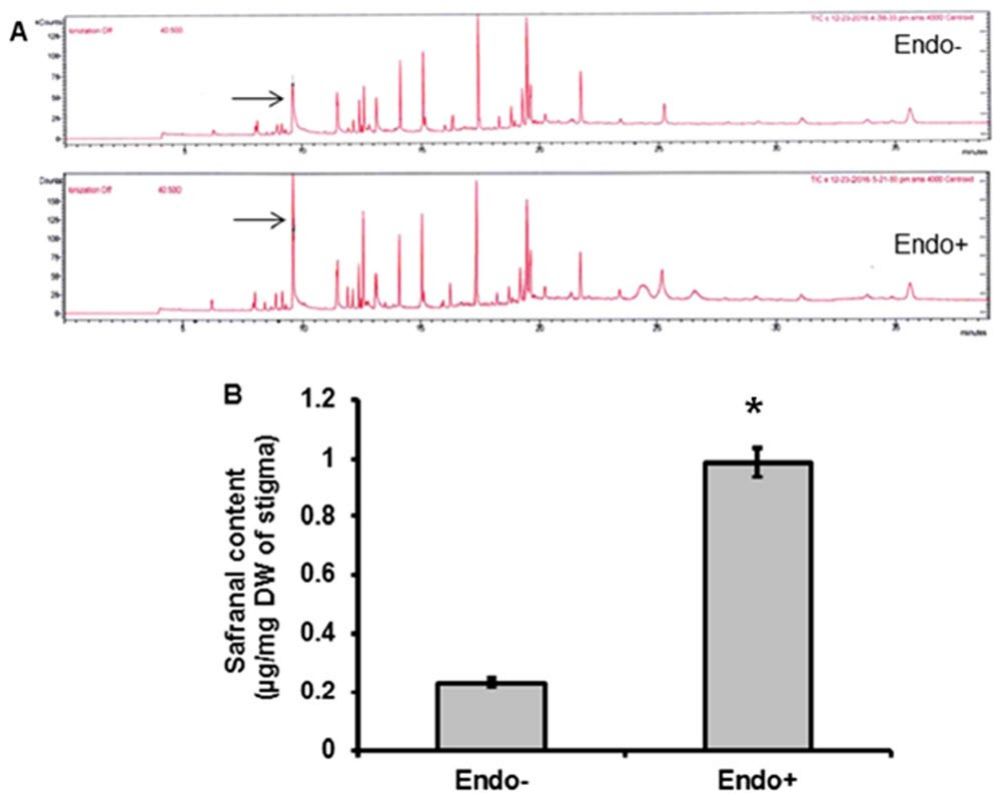
Effect of the CS10E4 on safranal content of saffron. (**A**) GC-MS chromatograms showing safranal peaks in endophyte treated (Endo+) and control (Endo−) plants (**B**) Bar diagram showing safranal content (µg/mg dry weight of stigma). Values are the means ± S.D. Comparisons among means were carried out using Student T test at a significance level of P ≤ 0.05 using GraphPad InStat tm software (V2.05).

Apocarotenoids are oxidative cleavage products of carotenoids and *Crocus* is known for being the only plant which produces apocarotenoids like crocin, picrocrocin and safranal in significant amounts. In the present study we evaluated effect of *M. alpina* CS10E4 on crocin and safranal content in *Crocus* stigmas (commercially called as saffron). The HPLC quantification of crocin showed around 2 fold increase in endophyte treated plants as compared to the control ones (Fig. 2). We also quantified safranal content by GC-MS and found around 4 fold increase in endophyte treated plants as compared to the control ones (Fig. 3). These results demonstrate the potential of *M. alpina* CS10E4 for enhancing the apocarotenoid content of *Crocus*.

**Figure 4:**
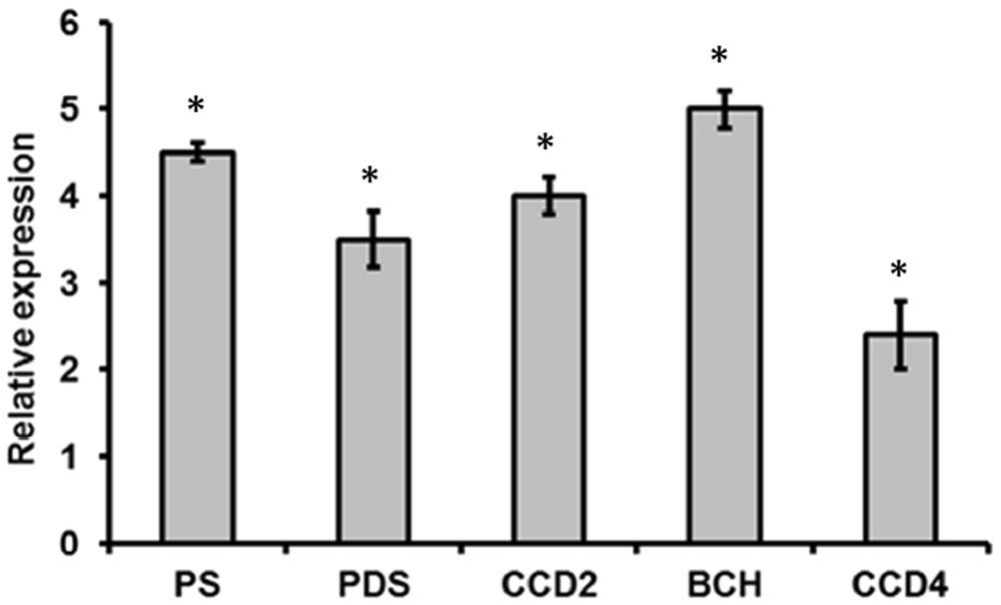
Effect of endophyte (CS10E4) on expression of genes involved in apocarotenoid biosynthesis. Transcript abundance of *phytoene synthase* (*PS)*, *phytoene desaturase (PDS)*, *betacarotene hydroxylase* (*BCH)*, *carotenoid cleavage dioxygenase 2 (CCD2)*, *carotenoid cleavage dioxygenase 4* (*CCD4)* was analyzed. Data are shown as relative expression levels of endophyte treated plants (Endo+) in comparison to the endophyte free control (Endo−) plants. 18 S was used as the internal reference gene. Data are means ± SD from three biological replicates. *Represents significance at P ≤ 0.05 using GraphPad InStat tm software (V2.05).

In order to gain an understanding about the mechanism of endophyte induced enhancement in apocarotenoid production, we performed expression profiling of some key apocarotenoid biosynthetic pathway genes. We checked the expression of genes encoding enzymes of major pathway steps. The results showed that the expression of phytoene synthase (PSY) which drives the rate limiting step of carotenoid/apocarotenoid pathway showed more than 4.5 fold induction in endophyte treated plants. Further, beta carotene hydroxylase (BCH) which is responsible for converting beta carotene into zeaxanthin, the actual precursor of crocin, picrocrocin and safranal, showed 5 fold induction in endophyte treated plants. We also checked the expression of two isoforms of carotenoid cleavage dioxygenase genes (*CsCCD2* and *CsCCD4*). The *CsCCD2* gene which cleaves zeaxanthin into crocin and picrocrocin showed 4 fold induction, whereas *CsCCD4* which cleaves beta carotene into beta ionone and cyclocitral showed 2.4 fold induction (Fig. 4).

### Effect of *M. alpina* CS10E4 on *Crocus* stress tolerance

One of the prominent causes of yield loss in saffron is the corm rot disease caused by fungal pathogen *F. oxysporum*. We investigated the antagonistic activity of the *M. alpina* CS10E4 against *F. oxysporum* R1 isolate in dual culture plate assay and it was observed that the percent growth inhibition of *F. oxysporum* R1 isolate was 53.5 ± 3.4% (Table S2). To confirm the relevance of dual culture inhibition of the pathogen by the endophyte, we performed *in vitro* and *in vivo* assays in which *Crocus* corms were treated with endophyte only (E), pathogen only (P) and first with the endophyte followed by the pathogen (E + P). The results showed that there were negligible disease symptoms in corms treated with endophyte only. The corms which were treated first with the endophyte and then infected with pathogen showed lesser disease severity than the ones treated with pathogen only (Fig. S3A). In all the cases, disease severity index (DI) was calculated which was high (2.7 and 2.9 in case of *in vitro* and *in vivo* experiments, respectively) for corms inoculated with pathogen only as compared to the corms which were inoculated with endophyte before the pathogen treatment. In the latter case DI was only 1.3 and 1.5 in case of *in vitro* and *in vivo* experiments, respectively. The difference in the DI values was significant at p < 0.05 (Table 3). These results suggest that *M. alpina* CS10E4 has a significant effect on reducing corm rot in *Crocus*.

**Table 3:** Calculation of disease severity index in *Crocus* plants.

Treatments	Disease severity	
	*In vitro* experiment	*In vivo* experiment
C	0	0.2
E	0	0.2
P	2.7^a^	2.9^a^
E+P	1.3^b^	1.5^b^

Where, C stands for control (endophyte free plants), E stands for only endophyte (CS10E4) treatment, P stands for only pathogen (*F. oxysporum* R1) treatment, E+P stands for pathogen treatment after 3dpi of endophyte treatment. Analysis of variance (ANOVA) of disease severity among the four treatments was tested by Bonferroni test at a significance level of p ≤ 0.05 using GraphPad InStat tm software (V2.05). ^a^Are means significantly different from C and E at probability level (p < 0.05) and ^b^means significantly different from P at probability level (p < 0.05).

### *M. alpina* CS10E4 induces stress tolerance through jasmonic acid pathway

*M. alpina* species is known to produce arachidonic acid (AA) which acts as a signaling molecule in biotic stress pathways. In order to investigate the involvement of AA in *Mortierella* induced resistance to *Fusarium* rot in *Crocus* corms, we did GC-MS of *M. alpina* CS10E4 extract and the results showed that the endophyte produces AA in significant amounts (Table S3). To prove the involvement of AA, we treated *Crocus* corms with AA, pathogen only and first with AA followed by pathogen. The corms which were treated first with the AA and then infected with pathogen showed lesser disease symptoms than the ones treated with pathogen only (Fig. S3B). These results were similar as were obtained with endophyte treatment (Fig. S3A). This suggested that decrease in corm rot symptoms and severity was due to AA released by endophyte.

**Figure 5:**
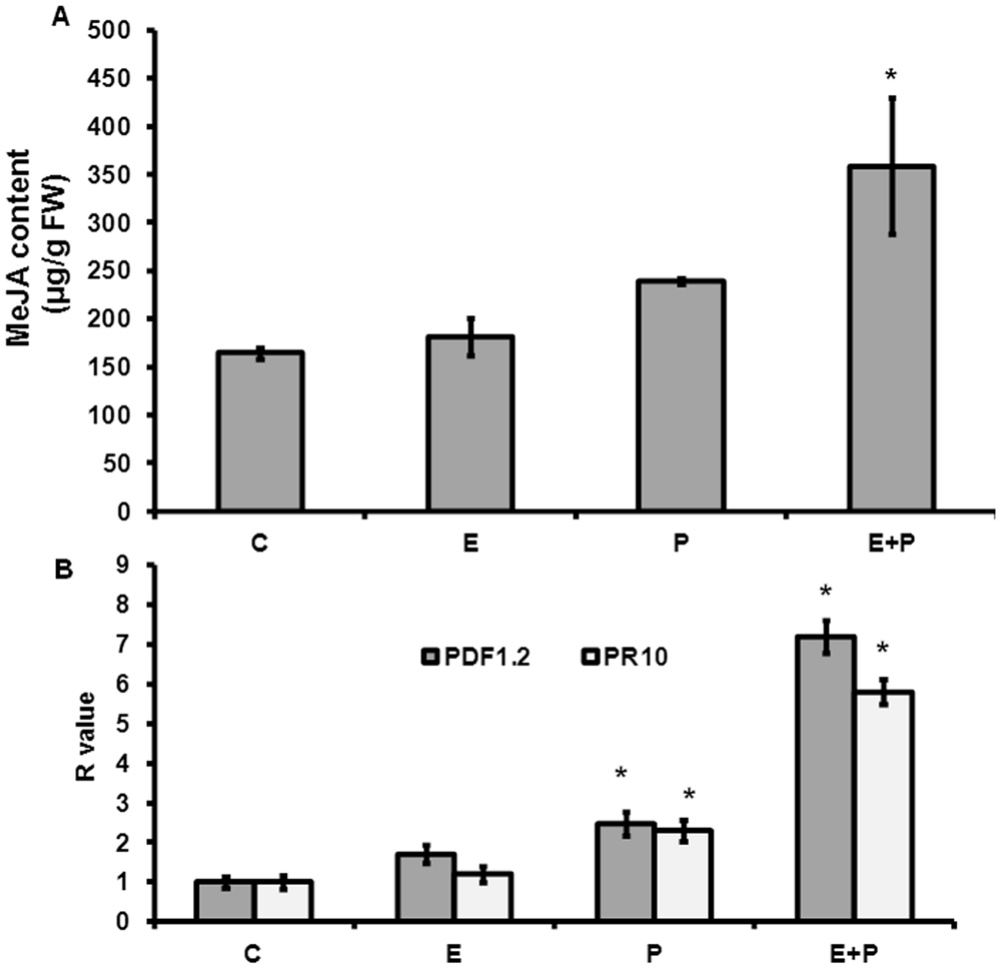
Quantification of jasmonic acid (JA) content and expression of JA responsive genes in *Crocus* corms. (**A**) Bar diagram shows JA content (µg/g fresh weight) in *Crocus* corms for all the four treatment conditions. (**B**) Transcript abundance of *PDF 1.2* and *PR10* genes in *Crocus* corms. where, C means control (endophyte free plants), E means only endophyte (CS10E4) treatment, P means only pathogen (*F. oxysporum* R1) treatment and E + P means pathogen treatment 3dpi of endophyte. 18 S was used as the internal reference gene. Data are means ± SD from three biological replicates. *Represents significance at P ≤ 0.05 using GraphPad InStat tm software (V2.05).

Further, it is known that AA mediates enhanced tolerance to pathogen stress through jasmonic acid^16^. We, therefore, quantified JA content using HPLC in *Crocus* corms treated with endophyte only (E), pathogen only (P) and endophyte followed by pathogen (E + P). The results showed that JA content in E, P and E + P treated corms was 181.1, 239.3 and 358.82 ngJA/g FW, respectively (Figs 5A and S4). Thus JA was much higher in case of corms first treated with endophyte followed by pathogen infection. In order to further confirm involvement of JA, we checked expression of two JA responsive genes and observed that both these genes showed higher expression in corms first treated with endophyte followed by pathogen as compared to corms treated with pathogen or endophyte alone (Fig. 5B). These results indicate involvement of JA in mediating defense pathways in endophyte treated corms.

## Discussion

Saffron, the dried stigmas of *Crocus*, constitutes an important spice and coloring agent in food and cosmetic industries. In addition to the organoleptic properties, the metabolite content of saffron particularly the apocarotenoids display tremendous therapeutic properties^9^. Moreover, saffron being the sole source of apocarotenoids like crocin, picrocrocin and safranal makes it an important crop to study. Because of its sterile nature, the crop production and productivity have been stagnant since many years. Further, the corm rot disease caused by *Fusarium oxysporum* is the most destructive disease in saffron known to devastate the crop fields leading to significant yield loss^18^. Therefore, an integrated approach aimed at improving saffron production in terms of crop yield and apocarotenoid content should be made an immediate focus. Since endophytes are known to have potential of promoting growth and stress tolerance in host plants and enhance secondary metabolite production^1, 19^, they present an important alternative for improvement of *Crocus* production and quality. In this context and in order to get an insight about the microbiome of *C. sativus*, previously work aimed at isolation of fungal endophytes from *Crocus* corms was done in our laboratory^15^. One of the endophytes isolated was *M. alpina*, which was the only zygomycete associated with *Crocus* corm. It showed antimicrobial activity and growth promoting properties^15^ and unlike other genera of zygomycetes, *M. alpina* is not known to cause any disease in plants, animals, or humans^20^. *M. alpina* has been extensively studied for the production of poly unsaturated fatty acids (PUFA), but very less work has been done on *M. alpina* vis-a-vis plant interaction. The present study was aimed at understanding the interaction of this endophyte (*M. alpina* CS10E4) with the host plant (*C. sativus*).

In order to investigate the role of *M. alpina* CS10E4 on *Crocus* growth and metabolism, we developed the host-endophyte association by inoculating this fungus into endophyte free *Crocus* corms and then planting them in the green house. The presence and hence successful association was confirmed by re-isolating the fungus from the corms after different time intervals and at different stages of development. In order to have a comprehensive picture of the effect of this endophyte on *Crocus*, we studied different growth parameters of control and endophyte treated plants. In *Crocus*, corm characteristics have a direct influence on flowering process. Flowers are formed from apical buds of the underground corms and the flower formation is directly related to corm size and biomass^18^. We observed that the endophyte significantly increased corm size and biomass and also the number of apical buds sprouting per corm (Table 1). Therefore, the endophyte enhances the probability of flower formation by improving the corm characteristics which have a direct bearing on flower production. Further, the main source of apocarotenoid production in *Crocus* is stigma. Therefore, it was interesting to know if endophyte had any effect on stigma features. We measured length and dry weight of stigmas collected from control and endophyte treated plants. While there was no significant change in the length of stigmas, their dry weight showed an increase in case of endophyte treated plants (Table 1). This was a very important observation and may be partly attributed to the fact that *M. alpina* CS10E4 is a proficient producer of IAA which is known to play role in stigma development during flower formation in many plants like *Arabidopsis* ^21^. Endophytes are also known to increase photosynthetic efficiency of plants^5^. In this context we also measured chlorophyll content of endophyte treated and control plants and observed a significant increase in chlorophyll content of endophyte treated plants (Table 1). This is indicative of higher photosynthetic efficiency of these plants.

The focus of our study was to know about the effect of endophytes on *Crocus* secondary metabolism. Our results indicated a significant increase in total carotenoid, phenolic and flavonoid content in endophyte treated plants (Table 2). Next we investigated the effect of endophytes on specialized metabolites of *Crocus* like crocin and safranal. We observed around 2 and 4 fold increase in crocin and safranal content respectively in endophyte treated plants compared to control ones (Figs 2 and 3). Previously increase in secondary metabolite content by endophytes has been reported from many other plants. For example, fungal endophytes of *Catharanthus roseus* were shown to enhance vindoline content^5^. Also endophytes of poppy were shown to modulate production of benzylisoquinoline alkaloids^6^. In order to gain an understanding about the mechanism by which endophytes modulate secondary metabolic pathways, we checked the expression of key pathway genes of apocarotenoid biosynthesis and the results indicated significant increase in gene expression in endophyte treated plants (Fig. 4). Phytoene synthase, the rate limiting enzyme showed 4.5 fold increase. Further BCH which is involved in formation of zeaxanthin, the immediate precursor of *Crocus* apocarotenoids showed 5 fold increase. This might lead to diverting metabolic flux towards increased production of crocin, picrocrocin and safranal. The elevated levels of gene expression might be because of the involvement of phytohormones. *M. alpina* CS10E4 produces IAA and releases arachidonic acid which in turn induces production of jasmonic acid (JA). Both IAA and JA are shown to have positive influence on expression of apocarotenoid biosynthetic pathway genes in *Crocus* ^8^. The manipulation of the host metabolism by the endophytes has been reported in recent times by various workers^5, 19, 22^. However understanding the dynamics of these endophyte-induced plant metabolome shifts will be a major focus of future research.

Corm rot is reported to be the most destructive disease in many *Crocus* growing areas resulting in considerable yield loss^18^. The corm rot is being managed by using chemical fungicides; however, the deleterious impact of these chemicals on the environment as well as humans is now well established^23^. These chemicals also affect the beneficial microflora associated with the plant and put selection pressure for evolution of resistant pathotypes^24^. As a result, biological control is gaining importance for integrated pest/disease management. The strategy of using antagonistic endophytes as biocontrol agents has been already reported^25–27^. We also investigated role of *M. alpina* CS10E4 in imparting tolerance to corm rot fungus. To begin with, we did dual inhibition assay which showed that *M. alpina* CS10E4 reduced the growth of *F. oxysporum* by 53.5% (Table S2). We went a step further and did *in vitro* and *in-vivo* assay in which corms were inoculated with endophyte only, pathogen only and first with endophyte followed by pathogen (Fig. S3A). The endophyte was able to reduce the disease severity by more than 50% in corms treated first with endophyte and subsequently with pathogen (Table 3).

Further, we wanted to know about the mechanism by which the endophyte inhibits corm rot in *Crocus*. *M. alpina* is known for producing polyunsaturated fatty acids (PUFAs) including arachidonic acid (AA)^28^. AA acts as evolutionary conserved signaling molecule which modulates stress signaling pathways^16^. Therefore we were interested to know if *M. alpina* CS10E4 also produces AA. The GC-MS results of the fungal extract showed presence of AA (Table S3), therefore, confirming that the endophyte might release AA in plant tissue. To know whether corm rot resistance in endophyte treated corms is actually because of AA, we set another experiment where corms were treated with AA followed by inoculation by pathogen causing corm rot. We observed that disease severity was less in AA treated corms than the control ones (Fig. S3B). Thus these results mimicked those obtained with endophyte treated corms which suggests that the disease resistance is due to release of AA in endophyte treated corms.

Most of the plants do not produce AA but are known to respond to AA signal by accumulating higher levels of jasmonic acid (JA)^16^. Thus AA induces defense response by acting via JA pathway. We quantified JA in the *Crocus* corms which were given different treatments viz. endophyte alone, pathogen alone, and endophyte followed by pathogen. It was observed that significantly higher JA content was accumulated in corms treated with CS10E4 followed by pathogen which was further confirmed by higher expression of JA responsive genes (Fig. 5). It may be because of AA released by endophyte which in turn induced JA production. This signifies that less disease index in endophyte treated corms in response to corm rot fungus was because of enhanced accumulation of JA. Since JA acts as a defense signal, it might have resulted in inhibition of pathogen growth in endophyte treated corms as compared to the control ones. This suggests that *M. alpina* CS10E4 induces resistance to corm rot by releasing AA which in turn leads to enhanced accumulation of JA.

## Methods

### Plant material and growth conditions

*C. sativus L*. plant material used for the present study was grown in experimental farm at Indian Institute of Integrative Medicine, Srinagar, Jammu and Kashmir, India (longitude: 34^◦^ 5^′^ 24^′′^ N; latitude: 74^◦^ 47^′^ 24^′′^ and altitude 1585 masl). Plants were grown in a greenhouse in natural photoperiod and light intensity. The voucher specimen under accession number 22893 was submitted at Janaki Ammal Herbarium, CSIR-IIIM, Jammu.

### Isolation and cultivation of endophytic fungus

The endophytic fungus was isolated from surface sterilized *Crocus* corms as described previously^15^. The colony texture, topography, color, colony growth pattern, and margin along with its microscopic structures like hyphal characteristics were recorded. The voucher specimen has been deposited in The Microbial Type Culture Collection and Gene Bank (MTCC), CSIR-IMTECH, Chandigarh, India, bearing no. MTCC 25134.

### DNA extraction, gene fragment amplification, sequencing and phylogenetic analysis

Genomic DNA was extracted from fungal mycelia using a modification of a previously described protocol^29^. Phylogenetic analysis of the endophyte was carried out by the acquisition of the ITS1-5.8S-ITS2 ribosomal gene sequencing. The ITS region of the fungal ribosomal DNA (ITS1-5.8S-ITS2) was amplified using universal primers ITS5 (5′-GGAAGTAAAAGTCGTAACAA-3′) and ITS4 (5′-TCCTCCGCTTATTGATATGC-3′)^30^. The phylogenetic positioning of the endophyte was inferred by using the Maximum Likelihood method based on the Tamura-Nei model^31^. Evolutionary analyses were conducted using MEGA6^32^. The ITS sequence of the *M. alpina* CS10E4 was deposited in GenBank with accession number KR135146.

### Growth pattern and plant growth promoting properties of the endophyte CS10E4

The endophyte CS10E4 was investigated for its growth pattern and time course indole acetic acid (IAA) production as described previously^15^. Siderophore production was detected using the Chrome Azurol S (CAS) agar plates^33^. Orange halos around colonies on blue agar indicated siderophore excretion.

### Endophyte inoculum preparation, treatment and planting of *Crocus* corms

The endophyte CS10E4 was cultured in potato dextrose broth (PDB) in shaking incubator at 25 °C for 14 days. The culture broth was filtered using cheesecloth to separate the mycelia, followed by impregnation of autoclaved carrier material (Agropeat + vermiculite) with the fully grown microbial broth. Up-scaling of endophyte formulation was done by continuously adding the autoclaved carrier material under sterile conditions.

In order to obtain endophyte free corms, fungicide bavistin (containing carbendazim 50% W.P, BASF India Limited) and bactericide K cycline (containing streptomycin sulfate 90% w/w and tetracycline hydrochloride 10% w/w) treatment was given to the corms for 24 hours duration. The corms were then washed with distilled water several times and allowed to dry. In order to confirm absence of any inherent endophytes, the imprints of the corm section cuttings after surface sterilization were put on PDA plates. The absence of any microbial growth confirmed that the corms were endophyte free.

For endophyte treatment, the surface sterilized corms were added to the endophyte formulation 3 days prior sowing. The endophyte was re-isolated from the corms at different time points to testify Koch’s postulate. A set of control was also used in which corms were added to the autoclaved carrier material with no microbial mass. Plantation of corms was done on raised bed/plots of 1 m^2^ geometry for each treatment.

### Measurement of growth and physiological parameters

To assess the effect of endophyte CS10E4 on growth of the host plant, various morphological parameters were recorded viz. total biomass, size of corms, number of apical buds sprouting per corm, number of adventitious roots per plant, length of adventitious roots, dry weight of stigma, length of stigma, and number of flowers. Total chlorophyll was calculated using the method described previously^13^.

### Measurement of total phenolic, flavonoid and carotenoid content

The extract preparation from *Crocus* flowers harvested from endophyte treated and control plants were done as follows. 1 g of *Crocus* flower tissue was crushed using liquid nitrogen followed by adding 5 ml of 80% methanol at 40 °C by continuous stirring for 8 h. This was repeated thrice; extracts were pooled, filtered, and dried using rotary vacuum concentrator and lyophilized. The lyophilized extracts were finally dissolved in methanol and used for measurement of total phenolic and flavonoid content. Total phenolic content was measured using the Folin–Ciocalteu reagent method^34^. The total phenolic content was expressed as mg of gallic acid equivalent (GAE) per gram of dry weight of the sample. Total flavonoid content was determined using the aluminum chloride colorimetric method^35^. The flavonoid content was calculated from the calibration curve and expressed as quercetin equivalents (mg of QUE per gram of dry weight).

Total carotenoid content was measured as described by^36^, with some modifications. 1 g of flower from endophyte treated and control plants were ground to powder in liquid nitrogen. The powder was homogenized in 5 ml ice-cold 80% acetone (Qualigens) and centrifuged at 10,000 rpm for 5 min and the supernatant obtained was transferred to a falcon tube containing 10 ml of petroleum ether. The acetone was removed by slowly adding ultrapure water and the aqueous phase was discarded. The procedure was repeated until no residual solvent remained. The extract was then transferred to a flask and anhydrous sodium sulfate was added. Using petroleum ether the volume was made up and the samples were read at 450 nm. The total carotenoid content was calculated as:

1 $${\rm{Carotenoid}}\,\mathrm{content}\,(\mu g/g)=\frac{\mathrm{Absorbance}\,\times \mathrm{Volume}\,\mathrm{of}\,\mathrm{extract}\,(\mathrm{ml})\times {\mathrm{10}}^{{\rm{2}}}}{{\rm{EC}}\times {\rm{sample}}\,{\rm{weight}}\,(g)}$$

EC = 2592 (β-carotene extinction coefficient in petroleum ether).

### Sample preparation and HPLC analysis

Crocin from *Crocus* stigma was extracted as described^37^. Briefly, 100 mg dried stigma was crushed in liquid nitrogen. The powder was homogenized in 2 ml Tris–HCl (50 mM, pH 7.5 containing 1 M NaCl), and incubated for 10 min on ice. This was followed by addition of 1 ml of chloroform. The extract was then incubated on ice for an additional 10 min. Centrifugation at 3000 g for 5 min at 4 °C was done to separate the phases. The lower chloroform phase was evaporated and the dried residues were stored together with the upper aqueous phases at −80 °C until high-performance liquid chromatography (HPLC) analysis.

Jasmonic acid (JA) was extracted from *Crocus* corms as described^38^. Briefly, 1 g of corm tissue was crushed in liquid nitrogen and the powder was extracted with MeOH-H_2_O-HOAc (90:9:1). The extraction was repeated twice and the pooled supernatant was dried. The dried residue was resuspended in 0.05% HOAc in H2O-MeCN (85:15, V/V) followed by filtration using 0.45 µm millipore filters and the filtrate was used for HPLC analysis.

The HPLC apparatus comprises of UFLC (Shimadzu) instrument equipped with pump (LC-20AD), autosampler (SIL-20AHT), column oven (CTO-10ASvP) and PDA detector (SPD-M20A). The separation was achieved on C_8_ Chromolith^®^ Merck (100 mm × 4.6 mm, 5 μm). For crocin quantification, the mobile phase consisted of 90:10 (V/V) mixture of water with 0.1% formic acid (A): acetonitrile (B) used in a linear gradient flow with a flow rate 1 ml/min. The column temperature was set at 40 °C initially. For JA quantification, the mobile phase consisted of 30:50:20 (V/V/V) mixture of water (A): acetonitrile (B): methanol (C) at a flow rate 1 ml/min. The column temperature was set at 35 °C initially. The analysis was performed with LabSolutions software (Schimadzu). The pure compounds of crocin and methyl jasmonate used as standards were obtained from Sigma-Aldrich.

### Sample preparation and GC/MS analysis

For safranal quantification, extract was prepared by crushing 100 mg of dried stigma in liquid nitrogen. The powder was homogenized in 1 ml of chloroform (Himedia). The extract was then incubated on ice for 10 min. Centrifugation at 5000 g for 5 min separated the supernatant, which was pooled and allowed to evaporate. The dried residue was dissolved in ethyl acetate (Burgyone) and used for GC-MS analysis.

For analysing the VOC’s from fungal extract, the extraction was performed according to the procedure described by^39^ with minor modifications. The endophyte CS10E4 was cultured in PDB for 15 days under shaking conditions at 25^◦^C. Mycelia were separated by filtration using cheesecloth followed by washing with distilled water. Mycelia were added to 50 ml of HCl (1 N) and kept in boiling water for 1 h followed by extraction with chloroform: methanol (2:1) solvent system and the extraction were repeated three times. The pooled supernatant was concentrated and analysed by gas chromatography.

The GC-MS analysis of the samples was performed using a Varian- 3800 GC, equipped with a CP-Sil-8 capillary column (30 m × 0.32 mm × 0.25 µm film thickness), and a mass spectrometer 4000. The carrier gas was helium, at a flow rate of 1 ml/min. Column split ratio was 1:50, column temperature 60 °C for 5 min, 250 deg @ 3 deg/min, and hold for 7 min. For GC–MS detection, an electron ionization system was used with ionization energy of 70 eV. The identification of the compounds produced by the endophyte was made via library comparison using National Institute of Standards and Technology (NIST) database. Only those compounds showing a match of 75% or more were recorded.

### Gene expression analysis using quantitative real time PCR

For real time PCR, total RNA was extracted from flowers of endophyte treated and control plants using TRIzol reagent (Invitrogen) and used for cDNA synthesis by reverse transcription kit (Fermentas) following manufacturer’s instructions. Real time PCR was performed in triplicates in Mx3000 P QPCR System (Stratagene). The reaction was carried out in a total volume of 10 μl which consisted of 5 μL of 2X SYBR Green Master Mix, 0.2 μM gene specific forward and reverse primers and 100 ng of template cDNA. The cycling parameters were 95 °C for 10 min, followed by 40 cycles of 95 °C for 15 s, 52 °C for 30 s, 72 for 1 min followed by melting curve program of 95 °C for 15 s, 55 °C for 30 s, 72 for 1 m. 18 S rRNA gene was used as an endogenous control to normalize the data. The control was used as the calibrator and relative gene expression was analyzed by the comparative Ct method (2^−ΔΔCt^ method)^40^. The sequence of the primers used in this study is given in Table S1.

### *In vitro* and *in vivo* assays for measuring disease severity index

Effect of endophyte on *Crocus* corm rot was studied under *in-vitro* and *in-vivo* conditions as described previously^15^. Briefly, for *in vitro* studies small injuries were made at the top of the sterilized corms with a sterile scalpel, and equal sized fungal plugs (approx. 2 mm) were put on it along with agar media to ensure fungal endophyte grow for successful infection. For *in vivo* studies surface sterilised corms were placed in paper cups containing autoclaved soil. Treatments were given in four different regimes, C, E, P, and E + P, where, C stands for control; E for endophyte (CS10E4); P for pathogen (*F. oxysporum* R1); E + P stands for pathogen treatment 3days post inoculation (dpi) of endophyte. Observations were recorded at 21 dpi for corms showing symptoms of corm rot and disease severity index (DI) was calculated^41^.

DI was calculated as follows:

2 $${\rm{DI}}=\frac{0({\rm{Hn}})+1({\rm{Xn}})+2({\rm{Yn}})+3({\rm{Zn}})}{{\rm{Total}}\,{\rm{number}}\,{\rm{of}}\,{\rm{plants}}}$$

where, Hn – number of healthy plants, Xn – number of plants having lesion in less than half the corm, Yn – number of plants having lesions in half the corm, Zn – number of plants having lesions in more than half the corm or complete corm.

### Statistical analyses

For growth parameters, 35 individual plants were analyzed from control and endophyte treatment as biological replicates. For the estimation of phenolics, flavonoids and carotenoids, and qPCR studies, three individual plants were analyzed independently as biological replicates. Technical replicates, to keep a check on analytical errors, were carried out for qPCR studies and quantification of crocin, safranal, and JA. Experiments were independently conducted and the values presented are the means ± SD. Comparisons among means were carried out using Student T test at a significance level of P ≤ 0.05 using GraphPad InStat tm software (V2.05).

For disease severity studies 12 individual corms were analyzed for each treatment (C, E, P and E + P). Analysis of variance (ANOVA) of disease severity among the four treatments was tested by Bonferroni test at a significance level of P ≤ 0.05 using GraphPad InStat tm software (V2.05).

## Electronic supplementary material

Supplementary data
