# Supplementary material for: Mortierella alpina CS10E4, an oleaginous fungal endophyte of Crocus sativus L. enhances apocarotenoid biosynthesis and stress tolerance in the host plant
Source: Sci Rep. 2017 Aug 17;7:8598. doi: 10.1038/s41598-017-08974-z (PMC5561177; doi:10.1038/s41598-017-08974-z)

Mortierella alpina (CS10E4), an oleaginous fungal endophyte of *Crocus sativus* L. enhances apocarotenoid biosynthesis and stress tolerance in host plant.

Zahoor Ahmed Wani[1,2], Amit Kumar[3], Phalisteen Sultan[1], Kushal Bindu[3], Syed Riyaz-Ul-Hassan[2,4]*, Nasheeman Ashraf[1,2]*

[1]Plant Biotechnology Division, CSIR-Indian Institute of Integrative Medicine, Sanat Nagar, Srinagar-190005, India

[2]Academy of Scientific and Innovative Research, CSIR-Indian Institute of Integrative Medicine, Canal Road, Jammu Tawi-180001, India

[3]Instrumentation Division, CSIR-Indian Institute of Integrative Medicine, Canal Road, Jammu Tawi-180001, India

[4]Microbial Biotechnology Division, CSIR-Indian Institute of Integrative Medicine, Canal Road, Jammu Tawi-180001, India

***Corresponding author:**

Nasheeman Ashraf
E mail: nashraf@iiim.ac.in
Phone number: +91 9797011714; Fax: +91-194-2441331

***Co-corresponding author:**

Syed Riyaz-Ul-Hassan
E mail: srhassan@iiim.ac.in
Phone number: +91 9419285434

Table S1: Sequence of the primers used in qPCR studies

Gene	Forward primer (5’---->3’)	Reverse primer (5’---->3’)
PS	TTC CCG TCA TGG GAA TTG CAC C	AAG GCC TGA TTG TGC AAG CTC
PDS	TAG CTG AGT CAT GGA ATA CTC	TTG CGC GGA GTG AGA AGT GC
BCH	ATGGGCCCCACAGGGAACCG	GGA GGG CCC TTT CGA GCT CAA
CCD4	ATCTCATCACCCGACACTCC	TTG GGT TGG GAC CGT TGC GG
CCD2	GTCAGTTGAGTTCTGCAGAGGT	TAGAGTAAAGTGGAATTCAAT
PDF1.2	GATGGTGAGAGTGGAGGGTCG	GCCGGCCTATTCTGGGCAAGGC
PR10	AGCACAGCCGTATCCGGTGG	CCTCCGTTGCTTGCTGCCTC
18S	ACGAAACCCCGGCGCAGTGGGC	TCGCTACGTTCTTCATCGATGCG

Table S2: Anatgonistic activity of *M. alpina* CS10E4 against common pathogens of *Crocus sativus* by dual culture plate assay.

Percentage (%) growth inhibition of common fungal pathogens by endophytic strain *M. alpina* CS10E4

Fusarium oxysporium (MTCC-1755)	Fusrium oxysporum R1	*Sclerotina* sp. (MTCC-7114)	*Fusarium solani* (MTCC-350)	*Colletotrichum capsici* (MTCC-2071)	*Geotrichum candidum* (MTCC-3993)	*Aspergillus fumigatus* (MTCC-343)
56.5±1.3	53.5±3.4	58.8±2.5	41.7±2.3	58.3±3.1	70.5±1.6	62±2.3

Table S3: Volatile organic compounds (VOCs) produced by *M. alpina* CS10E4 determined by GC/MS analysis.

Compounds	Amount (%)
Cyclotetradecane	0.364
2,4-Di-tert-butylphenol	0.413
10-Heneicosane	0.632
10-Heneicosane	0.692
Palmitic acid, methyl ester	13.38
Octadecatrienoic acid, methyle ester	3.536
Linoleic acid, methly ester	6.595
Oleic acid, methly ester	9.47
Stearic acid, methyl ester	11.561
Arachidonic acid, methyl ester	53.357

Fig. S1: CAS agar plate assay for detection of siderophore production by *M. alpina* CS10E4. Orange halos produced indicate siderophore production by the endophytic fungus.

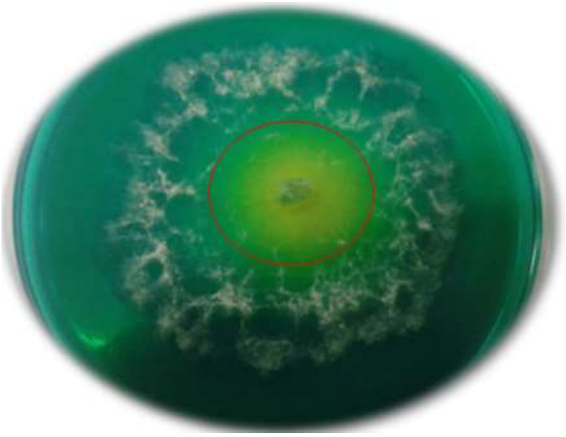

Fig. S2: Effect of endophyte (*M. alpina* CS10E4) treatment on morphological parameters of *Crocus* plant. Endo- means *Crocus* without endophyte treatment and Endo+ means *Crocus* with endophyte treatment.

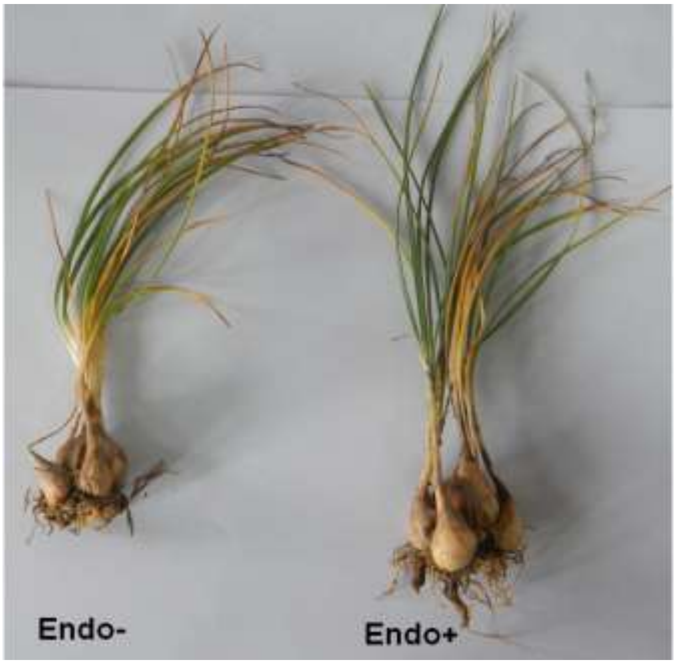

Fig. S3: *In vitro* study for measuring disease severity index in *Crocus* corms. (A) Where, C means control (endophyte free plants), E means only endophyte (CS10E4) treatment, P means only pathogen (*F. oxysporum* R1) treatment and E+P means pathogen treatment 3dpi of endophyte. (B) Where, AA means Arachidonic acid treatment at 50µM concentration, AA+P means pathogen treatment 3dpi of AA treatment, and P means only pathogen (*F. oxysporum* R1) treatment.

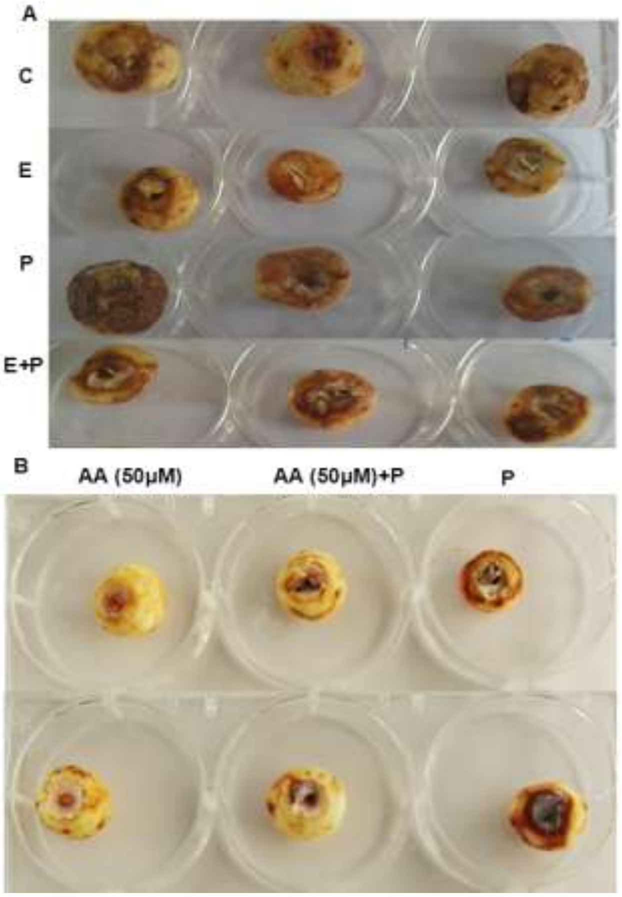

Fig. S4: Jasmonic acid (JA) content in *Crocus* corms. Chromatogram showing methyl jasmonate peaks in samples, where, C means control (endophyte free plants), E means only endophyte (CS10E4) treatment, P means only pathogen (*F. oxysporum* R1) treatment and E+P means pathogen treatment 3dpi of endophyte.

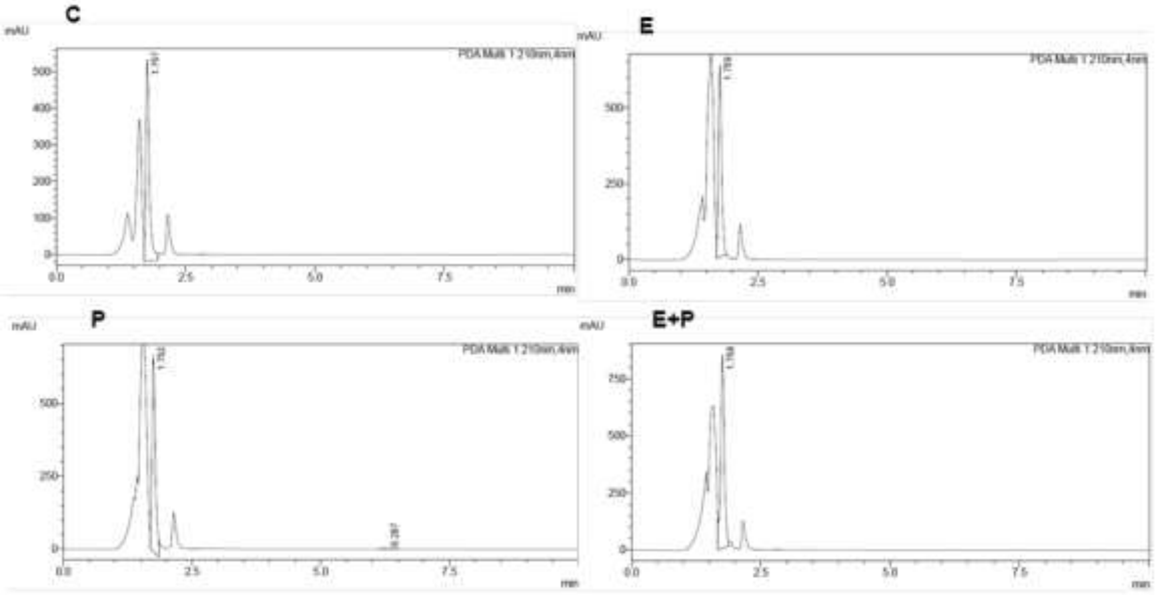

Supplement: Supplementary file 1 — Supplementary data [file 41598_2017_8974_MOESM1_ESM.pdf]
